# Foot Care Prioritization Among Health Care Providers Treating Diabetic Patients in Saudi Arabia: A Survey-Based Study

**DOI:** 10.7759/cureus.50798

**Published:** 2023-12-19

**Authors:** Rayan A Qutob, Osamah A Hakami, Layan Aldosari, Mohammad Alharfi, Raghad Y Alnader, Abdulaziz Alomar, Abdullah Alaryni, Abdullah Alghamdi, Eysa Alsolamy, Khalid Al Harbi, Yousef Alammari, Abdulwahed Abdulaziz Alotay, Mohammad A Alhajery, Abdulrahman Alanazi

**Affiliations:** 1 Department of Internal Medicine, College of Medicine, Imam Mohammad Ibn Saud Islamic University, Riyadh, SAU; 2 Department of Internal Medicine, King Abdullah Medical City in Holy Capital (KAMC-HC), Makkah, SAU; 3 College of Medicine, Imam Mohammad Ibn Saud Islamic University, Riyadh, SAU

**Keywords:** physicians, saudi arabia, healthcare, foot care, diabetes mellitus

## Abstract

Background: Diabetic foot disease (DFD) is a significant complication associated with diabetes, characterized by the potential for progressive amputation of specific foot segments or the entire lower limb in the absence of timely identification of infection and intervention. The aim of our research is to evaluate the degree of importance given to foot care by healthcare professionals who are responsible for treating individuals with diabetes in Riyadh, Saudi Arabia.

Methods: This cross-sectional study utilized an online survey previously validated in Australia. The mean foot care prioritization score was used to identify the dummy variable for binary logistic regression analysis, which was used to identify predictors of foot care prioritization.

Results: A total of 222 participants were involved in this study. Assessing for the risk of developing foot complications, visually inspecting feet for wounds, and providing or recommending footwear to prevent foot complications were the most commonly reported practices, accounting for 80.60% (n = 178), 76.10% (n = 169), and 75.20% (n = 167), respectively. The most commonly referred patients to a specialist tertiary multi-disciplinary foot care team were patients with ulcers in patients with absent foot pulses, ulcers with ascending cellulitis, and diabetic ulceration, accounting for 73.50% (n = 163), 71.60% (n = 159), and 66.70% (n = 148), respectively. The mean foot care prioritization score for the study participants was 54.1 (standard deviation: 11.7) out of 78 (69.4%), which demonstrates a moderately high level of foot care prioritization. Binary logistic regression analysis identified that healthcare professionals who are aged 35-44 years, those who have 5-10 years of experience, those who work at private hospitals, those who have a higher number of practice clinics per week, and those who have to manage a higher number of patients with diabetes in each clinic were more likely to prioritize foot care in their practices (p < 0.05).

Conclusion: Our study found that healthcare professionals in Saudi Arabia place a moderate degree of emphasis on foot care. Healthcare professionals falling within the age range of 35-44 years, possessing 5-10 years of experience, employed at private hospitals, overseeing a greater number of practice clinics weekly, and managing a greater number of patients with diabetes per clinic exhibited a greater propensity to prioritize foot care within their respective practices. Policymakers should consider the integration of continuous glucose monitoring technologies, the establishment of standardized foot screening protocols, and the implementation of targeted educational programs for healthcare professionals.

## Introduction

The worldwide prevalence of diabetes mellitus has significantly increased, with projections suggesting that the population of people affected by the condition will rise by 106 million by the year 2030, surpassing the current figure of 537 million [1]. Diabetes is accountable for around 6.7 million fatalities on an annual basis. Inadequate management of diabetes causes worsening glycemic control, which in chronic cases can involve multiple organs and cause complications [2-6]. Diabetic foot is a significant complication associated with diabetes, characterized by the potential for progressive amputation of specific foot segments or the entire lower limb in the absence of timely identification of infection and intervention. It is a medical condition in which a foot is affected by ulceration that is associated with neuropathy and/or peripheral arterial disease of the lower limb in a patient with diabetes. In order to mitigate the adverse consequences associated with diabetic foot conditions, it is imperative for individuals with diabetes to undergo periodic foot examinations aimed at identifying possible harmful indicators. These risk markers encompass a range of manifestations, including skin macules, ulcers, temperature fluctuations, disparities in blood pressure, bone deformities, as well as neurological and vascular abnormalities [7]. In addition, the training of healthcare providers can have a substantial impact on the mitigation of issues and the enhancement of patient care effectiveness [8].

Researchers conducted a study in 2022 to investigate the prevalence and potential risk factors of diabetic peripheral neuropathy (DPN), painful DPN, and diabetic foot ulceration (DFU) among individuals diagnosed with type 2 diabetes receiving secondary healthcare in Qatar, Kuwait, and the Kingdom of Saudi Arabia [9]. The study found that the prevalence of DPN was 33.3%. Among patients with DPN, a significant proportion (52.2%) were identified as being at risk of developing DFU, but a majority (53.6%) remained undiagnosed. The incidence of painful DPN was found to be significantly elevated at 43.3%, with a substantial proportion of cases (54.3%) remaining unidentified. The prevalence of DFU was observed to be 2.9%. The adjusted odds ratios for DPN and painful DPN exhibited an upward trend in relation to the longer duration of diabetes, obesity, suboptimal glycemic management, and hyperlipidemia. On the other hand, there was a negative association observed between higher levels of physical activity and the odds ratios of these disorders. The adjusted odds ratio for DFU was found to be elevated when co-existing with DPN, severe loss of vibration perception, hypertension, and vitamin D insufficiency. This research was the most extensive study conducted in the Middle Eastern region, shedding light on the considerable frequency of undetected DPN, painful DPN, and individuals susceptible to DFU among patients diagnosed with type 2 diabetes [9].

Numerous studies have been conducted to assess the level of awareness and adherence to foot care practices, as well as the prevalence of diabetic foot disease (DFD), among individuals with diabetes in the Middle East and North Africa (MENA) region [10,11]. In the year 2022, a cross-sectional study was undertaken in Egypt to examine diabetic foot care knowledge and practices related to microvascular problems among a sample of 100 individuals diagnosed with type 2 diabetes [11]. Only 25% and 24% of the participants exhibited satisfactory comprehension and implementation of diabetic foot care, respectively. The research group demonstrated a statistically significant positive correlation between knowledge and practice (p<0.001). The presence of microvascular issues contributes to the expansion of knowledge; however, its practical implications remain rather limited [11]. In 2015, Al-Kaabi et al. conducted a study in the United Arab Emirates to examine how illiteracy affects both diabetic complications and foot care. In this study, approximately 51% of the group consisted of individuals with limited literacy skills, who exhibited a decreased likelihood of engaging in foot care practices, comprehending foot risk factors, selecting suitable footwear, and participating in physical activities. Moreover, there was an increased susceptibility observed among the subjects to the onset of diabetes comorbidities, such as neuropathy (p = 0.027), diminished capillary refill time (p = 0.002), and decreased monofilament sensation. The statistical significance level for this analysis was determined to be p = 0.003 [12]. Another cross-sectional study conducted in 2020 examined the level of awareness and adherence to foot care practices among patients with diabetes mellitus attending primary care institutions in the Security Forces Health sector in Riyadh, Saudi Arabia. This study revealed that the majority of the patients, namely 58%, did not exhibit any foot-related complications. A significant proportion of patients, approximately 35.5%, experienced symptoms of numbness. A smaller percentage, 4.3%, reported a previous occurrence of a healed ulcer, while a little over 2.3% underwent a toe amputation. A majority of patients, specifically over 65%, reported a sense of confidence in comprehending self-care practices pertaining to their feet. There was no statistically significant disparity in knowledge levels observed between males and females. There were statistically significant differences seen between males and females in terms of self-care practices, specifically in the areas of frequent self-inspection and daily moisturization of the foot [13].

Conversely, several studies have examined the assessment of healthcare practitioners' knowledge, attitudes, and practices pertaining to diabetic foot disease. A noteworthy study conducted in Iran in 2022 sought to assess the effects of a diabetic foot workshop on the knowledge of nurses and physicians about the diagnosis and management of diabetic foot [14]. The researchers assessed the knowledge of a non-randomly selected group of nurses and physicians both before and after attending a diabetic foot workshop. The findings indicated a statistically significant enhancement in knowledge levels subsequent to the educational intervention, as evidenced by a mean post-test score that surpassed the pre-test score by over 20%. In summary, this research emphasizes the significance of educating healthcare professionals on the subject of diabetic foot care and the efficacy of multidisciplinary workshops in enhancing their knowledge and abilities [14]. A research article was published in 2022 that examined the level of knowledge, attitudes, and practices of primary healthcare providers in the Aseer region of Saudi Arabia in relation to the prevention and management of diabetic foot [15]. The research findings demonstrated a statistically significant and favorable association between the Knowledge, Attitudes, and Practices (KAP) ratings of primary healthcare providers in the Aseer region and physicians' specialty. The mean percentage of knowledge scores was found to be highest among family physicians compared to those with other specialties. Furthermore, it was observed that physicians with more than 10 years of experience in primary healthcare exhibited considerably higher knowledge scores [15].

A previous study by Mullan et al. evaluated the level of importance assigned to the management of DFD by primary healthcare practitioners in Australia in relation to other areas of diabetes care. The study findings indicated that primary care clinicians placed a greater emphasis on monitoring hemoglobin A1c (HbA1c) levels compared to doing preventative foot care exams and making recommendations to podiatry services. The prioritization of foot care assessments and referrals to podiatry was seen to occur mostly in response to the identification of a specific "foot concern." Furthermore, it was found that referrals to high-risk specialist foot podiatrists or services were prioritized for only 56% of participants in cases where significant amputation risk indicators were present. This study emphasizes the importance of primary healthcare professionals giving more priority to preventative foot care throughout the early stages of diabetes management in order to mitigate the occurrence of foot ulcers and amputations [16]. Therefore, the aim of our research is to evaluate the degree of importance given to foot care by healthcare professionals who are responsible for treating individuals with diabetes in Riyadh, Saudi Arabia. As of present, there has been no prior investigation that has employed this particular concept. The findings will offer healthcare professionals and policymakers valuable insights regarding educational initiatives and preventive actions aimed at healthcare providers and patients alike.

## Materials and methods

Study design

This study used an online survey that Mullan et al. [16] had previously validated in a cross-sectional study they conducted in Australia. The questionnaire was constructed on Google Form® and then distributed via online platforms to the targeted population of the study (inclusion criteria below) using a convenience sampling technique. The questionnaire link was distributed through social media websites (WhatsApp and Facebook). It was posted on relevant social media pages and groups. All participants were asked to provide their consent before participating in the study. This was highlighted in the cover letter of the questionnaire.

Study population

This study included Saudi and non-Saudi healthcare providers, including physicians (diabetologists, endocrinologists, internists, family physicians, and GPs), certified diabetes educators, and podiatrists practicing in Riyadh, Saudi Arabia, and treating adults with diabetes mellitus in the public and private sectors. We excluded healthcare providers who do not manage patients with diabetes, do not practice in Riyadh, or are not willing to participate in the study.

Questionnaire tool

The survey consists of three sections, with informed consent embedded at the beginning. The first section is about demographic data, which is modified to suit our study population. In the second section, participants were provided with five hypothetical clinical scenarios to evaluate their understanding of diabetic foot care and be aware of their top three prime concerns. The clinical scenarios involved consulting with five different patients: (i) a newly diagnosed patient with diabetes mellitus; (ii) a patient with a 20-year history of diabetes; (iii) a patient with diabetes who experiences a tingling sensation in the feet; (iv) a patient with diabetes who presented with a “small cut” on one foot; and (v) a patient diagnosed with diabetes who presented with the following findings: peripheral neuropathy, absent pedal pulses, and an ulcer 1 cm in diameter. Participants ranked their top three concerns in every situation. The last section focused on measuring the subjects' different approaches to the assessment and management of the patients using a rating scale of how often they do a certain process or procedure.

Foot care prioritization was assessed using 13 questions in a 6-point Likert scale format that ranged between “never and assigned a score of zero” and “always and assigned a score of 6." The maximum attainable score is 78. The higher the score, the greater the attitude towards foot care prioritization.

Questionnaire piloting and assessment

The questionnaire instrument was reviewed and verified by experienced endocrinologists. They assessed its clarity and readability, including its face validity and whether any questions were challenging to understand. The purpose of this review was to assess the lucidity, comprehensibility, and appropriateness of the questions, as well as to ascertain the acceptability of the content and detect any potential misconceptions. Prior to implementing the questionnaire on a larger scale, pilot research was undertaken with a limited number of participants (35 individuals) to evaluate its clarity; the findings verified its straightforwardness. The questionnaire items were subjected to a content validity assessment to ensure that they sufficiently addressed the pertinent topic of the study.

Sample size

The estimated sample size using the OpenEpi online tool is 287. It was based on the 2021 Ministry of Health report that the total number of physicians working in Saudi Arabia was around 50,000, with a confidence interval of 95% and an anticipated frequency of 75%.

Ethical approval

This study was accepted by the Research Ethics Committee at Al-Imam Muhammad Ibn Saud Islamic University, Riyadh, Saudi Arabia (reference number: 483/2023).

Statistical analysis

The collected data were analyzed using the Statistical Package for Social Science software, version 29 (IBM Corp., Armonk, NY). Categorical variables were presented as frequency and percentage. Continuous variables were presented as the mean and standard deviation (SD). The mean foot care prioritization score was used to identify the dummy variable for binary logistic regression analysis, which was used to identify predictors of foot care prioritization. An odds ratio with a 95% confidence interval was presented to present the findings of the regression analysis. A p-value of <0.05 is considered statistically significant.

## Results

A total of 222 participants were involved in this study. More than half of them were males (71.2%; n = 158) and aged 25-34 years (64.9%; n = 144). The vast majority of the participants were Saudis (84.7%; n = 188). Almost one-third of the study participants (34.2%) were general practitioners. Almost half of the participants (51.4%; n = 114) were residents. More than half of the participants (61.3%; n = 136) reported that they have had experience for less than five years. Almost half of the participants (48.6%; n = 108) reported that they work at primary healthcare centers. Almost one-third of the participants (31.1%; n = 69) reported that they work in a maximum of two practice clinics per week, and 37.4% (n = 83) of them reported that the number of diabetes patients in each practice clinic is around 6-10 patients. Further details on participants' demographic and practice characteristics are provided in Table 1.

**Table 1:** Participants' demographic and practices characteristics.

Variable	Frequency	Percentage
Gender		
Male	158	71.2%
Age categories		
25–34 years	144	64.9%
35–44 years	48	21.6%
45–54 years	22	21.6%
55–64 years	8	3.7%
Nationality		
Saudi	188	84.7%
Specialty		
General practitioner	76	34.2%
Family physician	53	23.9%
Internist	43	19.4%
Diabetologists	29	13.1%
Endocrinologist	9	4.1%
Podiatrist/foot specialist	8	3.6%
Certified diabetic educator	4	1.9%
Level of experience		
Consultant	21	9.5%
Fellow	25	11.3%
Senior registrar	30	13.5%
Registrar	32	14.4%
Resident	114	51.4%
Years of experience		
Less than 5 years	136	61.3%
5–10 years	43	19.4%
11–15 years	25	11.3%
16–20 years	14	6.3%
More than 20 years	4	1.8%
Place of practice		
Primary healthcare center	108	48.6%
Private hospital	29	13.1%
Diabetes care center (Ministry of health)	26	11.7%
Private polyclinic	22	9.9%
Specialty clinic at secondary hospital	19	8.6%
Specialty clinic at tertiary hospital	17	7.7%
Number of practice clinics per week		
2 or less	69	31.1%
3–4	66	29.7%
5–6	50	22.5%
7–8	33	14.9%
9 or more	4	1.8%
Number of patients with diabetes in each clinic		
5 or less	64	28.9%
6–10	83	37.4%
11–15	47	21.2%
16–20	17	7.7%
21 or more	11	5.0%

Healthcare professionals’ response to different clinical scenarios

Table 2 presents healthcare professionals’ responses to different clinical scenarios while managing patients with diabetes mellitus. The vast majority of the participants (83.3%; n = 185) reported that 50% or more of the time, they ask the patient with diabetes to remove the shoes and socks during the consultation. For patients newly diagnosed with type 2 diabetes who present to their clinics, the top three priorities in managing them were HbA1c review, medication review, and lifestyle education.

**Table 2:** Healthcare professionals response to different clinical scenarios

Variable				Frequency	Percentage
Physician priorities in managing patients with diabetes					
Priority	1. For a person newly diagnosed with type 2 diabetes presents to your clinic:	2. For a person with a 20-year history of diabetes presents to your clinic for the ﬁrst time:	3. For a person with diabetes presents to your clinic for the ﬁrst time and reports tingling in their feet:	4. For a person with diabetes presents to your clinic for the ﬁrst time and reports that they have a small cut on their foot:	5. If you conduct a full foot assessment on a person with diabetes and ﬁnd that they have evidence of peripheral neuropathy, absent pedal pulses and an ulcer of 1 cm in diameter:
HbA1c review (assessing blood glucose management)	55.9%	52.3%	29.7%	22.5%	23.0%
Medication review	9.0%	10.4%	5.0%	4.1%	6.8%
Blood pressure review and management	5.0%	4.1%	5.4%	7.7%	5.4%
Renal function assessment	4.1%	3.6%	4.1%	5.0%	0.9%
Lipid assessment	0.5%	2.7%	0.9%	0.5%	3.6%
Lifestyle education (diet/exercise/weight)	6.3%	3.2%	5.4%	4.1%	2.3%
Conduct full foot assessment (neurological, pulses, risk rating)	1.8%	2.3%	27.5%	17.6%	13.1%
Referral to podiatrist	0.5%	2.7%	1.4%	0.9%	3.2%
Eye examination	3.2%	0.9%	0.5%	0.5%	2.3%
Smoking assessment	0.9%	0.5%	0.5%	0.5%	0.5%
Self-management assessment	0.5%	0.0%	1.4%	2.7%	2.3%
Emotional/psychological health assessment	0.9%	0.5%	0.9%	0.9%	2.7%
Driving safety education	1.4%	0.0%	0.9%	0.5%	0.9%
Ongoing wound dressing in primary care	0.5%	0.9%	0.9%	12.6%	1.8%
Referral to diabetes educator	0.5%	0.9%	0.5%	0.9%	0.5%
Referral to general practitioner	0.5%	0.5%	0.0%	0.5%	1.4%
Referral to tertiary multidisciplinary diabetes service	0.5%	0.9%	0.0%	0.0%	2.3%
Referral to endocrinologist	0.9%	0.5%	0.9%	0.9%	1.4%
Referral to specialist tertiary diabetic foot clinic (multidisciplinary high risk foot service)	2.3%	0.5%	0.5%	4.5%	14.4%
Referral to vascular surgeon	0.9%	1.4%	2.3%	0.5%	3.6%

For patients with a 20-year history of diabetes present in their clinics for the first time, the top three priorities in managing them were HbA1c review, medication review, and blood pressure review and management. For patients with diabetes who present to their clinic for the first time and report tingling in their feet, the top three priorities in managing them were HbA1c review, blood pressure review and management, and conducting a full foot assessment. For patients with diabetes who present to their clinic for the first time and report that they have a small cut on their foot, the top three priorities in managing them were HbA1c review, conducting a full foot assessment, and ongoing wound dressing in primary care. If they conduct a full foot assessment on a person with diabetes and find that they have evidence of peripheral neuropathy, absent pedal pulses, and an ulcer of 1 cm in diameter, the top three priorities in managing them are HbA1c review, conducting a full foot assessment, and referral to a specialist tertiary diabetic foot clinic (Table 2).

For a highly functioning, independent 61-year-old working woman with poorly controlled type 2 diabetes but no previous complications present to your clinic who has a 1 cm × 2 cm ulcer, which appears to be deep, on the left plantar surface at the interphalangeal joint of the hallux. The noted 1.5 cm of surrounding cellulitis and their clinic notes reveal that she saw a podiatrist five months ago with no abnormalities reported by the consulting podiatrist at this visit. Besides, the woman reports that the ulcer has been there for a couple of weeks; it is not giving her any discomfort, and she has been washing it in salty water and putting bandages on it regularly. Furthermore, she is afebrile, with normal blood pressure and heart rate, and her most recent HbA1c was 8.1% (65 mmol/mol). The top three priorities were referral to other specialists, assessing and dressing the wound, and assessing the wound and referring to other healthcare professionals to dress the wound (Table 3).

**Table 3:** Healthcare professionals response to patient scenario number 6 For a highly functioning, independent 61-year-old working woman with poorly controlled type 2 diabetes but no previous complications present to your clinic. She has a 1 cm × 2 cm ulcer, which appears to be deep, on the left plantar surface at the interphalangeal joint of the hallux. You note 1.5 cm of surrounding cellulitis. Your clinic notes reveal that she saw a podiatrist 5 months ago with no abnormalities reported by the consulting podiatrist at this visit. The woman reports that the ulcer has been there for a couple of weeks. It is not giving her any discomfort and she has been washing it in salty water and putting bandaids on it regularly. She is afebrile, with normal blood pressure and heart rate and her most recent HbA1c was 8.1% (65 mmol/mol).

Priority	Percentage
Refer to other specialist (e.g. vascular surgeon, orthopedic surgeon, infectious diseases physician)	62.2%
Assess and dress the wound	32.0%
Assess the wound and refer to another healthcare professional to dress it (e.g., general practitioner, primary care nurse, or podiatrist)	29.3%
Conduct full foot assessment (neurological, pulses, risk rating)	27.5%
Continue regular review and wound care within primary care Refer to specialist diabetic foot clinic (multidisciplinary high-risk foot service)	27.0%
Swab the wound and send swab to pathology	24.8%
Refer to general practitioner for assessment and management	19.4%
Commence antibiotics	18.9%
Refer to other healthcare professional to conduct full foot assessment (e.g., general practitioner, certiﬁed diabetes educator, primary care nurse, private community podiatrist)	17.1%
Assess diabetes self-management, e.g., blood glucose levels	17.1%
Refer to hospital emergency department	5.0%
Refer to endocrinologist	4.1%

Diabetes-related foot assessment and management

Table 4 presents the study participants’ diabetes-related foot assessment and management. Assessing for the risk of developing foot complications, visually inspecting feet for wounds, and providing or recommending footwear to prevent foot complications were the most commonly reported practices given priority (answered often, very often, and always), accounting for 80.60% (n = 179), 76.10% (n = 169), and 75.20% (n = 167), respectively.

**Table 4:** Diabetes-related foot assessment and management

	Never	Very rarely	Rarely	Sometimes	Often	Very often	Always
When treating patients with diabetes, how often in the last 12 months did you do the following?							
Assess the risk of developing foot complications	0.9%	0.9%	4.5%	13.1%	21.6%	31.1%	27.9%
Inquire about previous foot ulcers and amputations	1.8%	3.2%	8.1%	13.5%	23.4%	26.1%	23.9%
Inspect feet for structural abnormalities	1.4%	4.5%	5.9%	17.1%	18.0%	28.4%	24.8%
Visually inspect feet for wounds	1.8%	4.1%	7.7%	10.4%	18.9%	31.1%	26.1%
Assess for neuropathy using 10 g monofilament	2.7%	4.1%	10.8%	23.0%	21.2%	27.9%	10.4%
Assess for neuropathy	0.9%	3.2%	10.8%	18.0%	27.5%	25.2%	14.4%
Palpate their foot pulses	1.4%	2.3%	6.8%	15.3%	27.9%	33.3%	13.1%
Perform an Ankle Brachial Index or Toe Pressure assessment	4.1%	8.1%	8.6%	11.7%	28.8%	28.4%	10.4%
Classify their risk of developing foot complications (low, intermediate, and high)	1.8%	5.4%	5.9%	17.6%	21.2%	31.1%	17.1%
Provide foot care education to prevent foot complications	2.7%	4.5%	4.1%	16.2%	25.7%	32.9%	14.0%
Provide or recommend footwear to prevent foot complications	2.3%	3.2%	5.0%	14.4%	30.2%	29.7%	15.3%
Recommend a review assessment annually for patients stratiﬁed as low risk	4.1%	3.6%	6.3%	17.6%	25.7%	30.2%	12.6%
Recommend a review assessment within 6 months for patient stratiﬁed as intermediate or high risk	3.2%	1.8%	5.9%	16.2%	19.4%	27.5%	26.1%

The majority of the participants (78.4%; n = 174) confirmed that they have access to and know how to refer patients with diabetes-related foot ulcers to a specialist tertiary multi-disciplinary foot care team. For those who do not have access, the most commonly reported management approaches were managing the patients at their practices and referring the patients to private vascular surgeons, accounting for 46.8% (n = 22) and 28.8% (n = 14), respectively (Table 5).

**Table 5:** Patients with diabetes-related foot ulcers referral

	Frequency	Percentage
Do you have access to and know how to refer patients with diabetes-related foot ulcers to a specialist tertiary multi-disciplinary foot care team (High Risk Foot Service)? (Yes)	174	78.4%
If you do not have access to and know how to refer patients with diabetes-related foot ulcers to a specialist tertiary multi-disciplinary foot care team (High Risk Foot Service), how do you manage these patients? (n= 48)		
Manage at your practice	22	46.8%
Refer to private vascular surgeon	14	28.8%
Refer to private community podiatrist	8	17.1%
Refer to general practice for management	4	7.2%

The most commonly referred patients to a specialist tertiary multi-disciplinary foot care team were patients with ulcers in patients with absent foot pulses, ulcers with ascending cellulitis, and diabetic ulceration, accounting for 73.50% (n = 163), 71.60% (n = 159), and 66.70% (n = 148), respectively (Table 6).

**Table 6:** Patients' referral characteristics How often in the last 12 months did you refer patients with the following conditions to a specialist tertiary multi-disciplinary foot care team?

	Never	Very rarely	Rarely	Sometimes	Often	Very often	Always
Diabetic ulceration	4.5%	2.7%	5.9%	20.3%	11.3%	24.3%	31.1%
Deep foot ulceration (probing a tendon, joint, or bone)	4.1%	4.5%	16.7%	9.9%	16.7%	32.4%	15.8%
Ulcer not reducing in size after 4 weeks	4.1%	14.4%	5.4%	9.9%	23.4%	26.6%	16.2%
Ulcers in patients with absent foot pulses	5.0%	3.6%	5.0%	13.1%	30.2%	26.6%	16.7%
Ulcers with ascending cellulitis	5.0%	5.0%	6.8%	11.7%	21.2%	38.7%	11.7%
Suspected Charcot’s neuroarthropathy	18.9%	5.9%	8.1%	8.6%	18.0%	26.6%	14.0%

Predictors of healthcare professionals’ foot care prioritization

The mean foot care prioritization score for the study participants was 54.1 (SD: 11.7) out of 78 (69.4%), which demonstrates a moderately high level of foot care prioritization. Binary logistic regression analysis identified that healthcare professionals who are aged 35-44 years, those who have 5-10 years of experience, those who work at private hospitals, those who have a higher number of practice clinics per week, and those who have to manage a higher number of patients with diabetes in each clinic were more likely to prioritize foot care in their practices (p<0.05; Table 7).

**Table 7:** Predictors of healthcare professionals’ foot care prioritization *p<0.05; **p<0.01; ***p<0.001

Variable	Odds ratio of foot care prioritization (95% confidence interval)	P-value
Gender		
Female (reference group)	1.00	
Male	1.68 (0.93–3.02)	0.085
Age categories		
25–34 years (Reference group)	1.00	
35–44 years	2.50 (0.124–5.04)	0.011*
45–54 years	1.03 (0.42–2.52)	0.952
55–64 years	6.17 (0.72–52.54)	0.096
Nationality		
Non-Saudi (reference group)	1.00	
Saudi	0.77 (0.36–1.64)	0.499
Specialty		
Diabetologist (reference group)	1.00	
Endocrinologist	1.05 (0.22–5.13)	0.949
Family physician	0.74 (0.29–1.90)	0.533
Internist	0.46 (0.17–1.21)	0.115
General practitioner	0.76 (0.31–1.86)	0.554
Podiatrist/ foot specialist	0.08 (0.00–0.70)	0.230
Certified diabetic educator	-	
Level of experience		
Consultant (reference group)	1.00	
Fellow	1.91 (0.58–6.30)	0.288
Senior registrar	1.96 (0.62–6.19)	0.251
Registrar	2.86 (0.90–9.08)	0.074
Resident	1.79 (0.68–4.71)	0.239
Years of experience		
Less than 5 years (Reference group)	1.00	
5–10 years	2.95 (1.40–6.23)	0.005**
11–15 years	2.03 (0.84–4.92)	0.116
16–20 years	2.86 (0.85–9.56)	0.088
More than 20 years	1.14 (0.16–8.35)	0.895
Place of practice		
Primary healthcare center (Reference group)	1.00	
Diabetes care center (Ministry of health)	1.86 (0.77–4.46)	0.166
Specialty clinic at secondary hospital	1.04 (0.39–2.77)	0.931
Specialty clinic at tertiary hospital	1.66 (0.59–4.68)	0.340
Private polyclinic	2.65 (1.01–6.96)	0.048*
Private hospital	3.05 (1.24–7.47)	0.015*
Number of practice clinics per week		
2 or less (reference group)	1.00	
3–4	5.85 (2.69–12.70)	<0.001***
5–6	19.62 (7.66–50.26)	<0.001***
7–8	24.12 (7.82–74.44)	<0.001***
9 or more	4.31 (0.55–33.49)	0.163
Number of patients with diabetes in each clinic		
5 or less (Reference group)	1.00	
6–10	5.04 (2.5–10.3)	<0.001***
11–15	5.77 (2.52–13.21)	<0.001***
16–20	3.87 (1.27–11.78)	0.017*
21 or more	12.18 (2.39–62.15)	0.003**

## Discussion

Diabetes mellitus often leads to a common and significant complication referred to as diabetic foot disease, which can result in both morbidity and mortality [17]. The primary pathologies contributing to diabetic foot disease encompass peripheral neuropathy, peripheral arterial disease, and infection. The consequences of these pathologies can result in various complications, including ulceration, Charcot foot, painful neuropathy, gangrene, and, in severe instances, amputation [17,18]. Consequently, it is imperative to conduct a thorough evaluation and promptly refer patients to a multidisciplinary team for the efficient management of diabetic foot issues [19]. Therefore, the aim of this study was to evaluate the prioritization of foot care among healthcare providers who treat individuals with diabetes in Riyadh, Saudi Arabia. Notably, no previous research has investigated this specific aspect in the region.

The findings of the study indicate that when patients are newly diagnosed with type 2 diabetes and seek treatment at healthcare facilities, the primary areas of focus for managing their condition are reviewing HbA1c levels to assess blood glucose control, evaluating medication usage, and providing education on lifestyle factors. A previous study in Australia found similar findings and reported lifestyle education, HbA1c review, and self-management assessment as the top three priorities of care among newly diagnosed patients with diabetes mellitus [16]. The emphasis on reviewing HbA1c levels as a primary aspect of care in this study aligns with earlier research, which found that prioritizing glycemic control takes precedence over preventive footcare measures and may hinder the provision of footcare [16]. The 2018 National Association of Diabetes Centers audit conducted in Australia revealed that 43% of them had not sought podiatry services within the past year [20]. This may be attributed to the prioritization of other aspects of diabetes care. Another study conducted in the United States provided support for this claim. Out of the participants, 65% agreed that other significant matters are prioritized above footcare [21]. It is imperative to implement a comprehensive approach to diabetes management that includes monitoring blood glucose levels, adhering to a suitable diet, engaging in regular physical activity, following prescribed medication regimens, and maintaining comprehensive health records [22]. In addition to other strategies for diabetes health management that can enhance lifestyle, improve physiological markers, reduce complication rates, and establish a positive feedback loop [23], continuous glucose monitoring and management achieve optimal disease management [24].

The findings of the study revealed that a significant percentage of the participants (83.3%) reported a consistent practice of requesting patients with diabetes to remove their shoes and socks in 50% or more of their consultations. This practice is deemed crucial as it serves as a screening measure for diabetic foot complications. It is well known that individuals diagnosed with diabetes mellitus are susceptible to various complications, including the development of diabetic foot ulcers. These ulcers affect approximately 15-25% of diabetic patients and are a leading cause of morbidity and mortality in this population. It is worth noting that 85% of diabetic amputees initially experience diabetic foot ulcers, which can progress to severe gangrene or infection [25].

In the course of our investigation, the prevailing methods utilized to assess the likelihood of foot complications were identified. These methods encompassed the evaluation of the risk of developing foot complications, the visual examination of feet for wounds, and the provision or suggestion of appropriate footwear to mitigate the occurrence of foot complications. These practices accounted for 80.60%, 76.10%, and 75.20% of the reported approaches, respectively. This was similar to the findings of a previous study in Australia, which identified that the three priorities for podiatrists to assess the likelihood of foot complications were inquiring about previous foot ulcers and amputations, visually inspecting feet for structural abnormalities, and visually inspecting feet for wounds [26]. It is imperative to emphasize the significance of regular foot screening in identifying individuals at risk of foot problems, such as peripheral neuropathy, foot deformity, peripheral arterial disease, and poor glycemic control. This screening process plays a crucial role in referring patients for appropriate management [27]. In the context of diabetes, various factors can contribute to the development of complications related to diabetic foot ulcers, which in turn can result in non-healing wounds. These factors include the depth of foot wounds, the presence of infection, and ischemia [28]. It is crucial for trained personnel to conduct regular foot examinations to identify risk factors for ulceration and provide appropriate foot-care management [29]. One effective intervention is the visual assessment of the foot, which is a simple and efficient procedure. However, it has been observed that such foot assessments are not consistently performed during every encounter in primary care settings [30]. On the other hand, the provision or recommendation of suitable footwear plays a significant role in preventing foot complications associated with diabetes [31]. When prescribed appropriately, footwear can effectively reduce pedal pressure and minimize the risk of foot ulceration in individuals with diabetes [32].

Furthermore, the findings of the study indicate that a significant proportion of the participants (78.4%) reported possessing the necessary access and knowledge to refer patients with diabetes-related foot ulcers to a specialized tertiary multi-disciplinary foot care team known as the High-Risk Foot Service. This availability of access and knowledge regarding the referral of patients to specialized teams is a positive development. It is essential to promptly refer individuals with newly developed foot ulcers to multidisciplinary diabetes foot care teams in order to improve the overall quality of patient care [33]. However, for individuals lacking access, the most frequently reported management approaches include managing patients at their practices, accounting for 46.8% of cases, and referring patients to private vascular surgeons, accounting for 28.8% of cases. Similarly, in India, it has been observed that physicians in independent diabetic foot clinics handle a variety of diabetic foot conditions at their own practices, utilizing various diagnostic tools and educational resources [34]. Conversely, referring patients to vascular surgeons has been found to be advantageous, as the integration of a vascular unit with community care has resulted in improved outcomes for patients with diabetic foot disease, including reduced rates of major amputations and foot surgeries [35].

In our research, the patients most frequently referred to a specialized tertiary multi-disciplinary foot care team were those with ulcers and absent foot pulses, ulcers with ascending cellulitis, and diabetic ulceration. These three groups accounted for 73.5%, 71.6%, and 66.7% of the referrals, respectively. The referred patients in these groups exhibited risk factors for ulceration, such as non-palpable pulses, insensate feet, significant calluses, deformed nails, a history of previous ulcers or amputations, tissue damage or signs of ulceration, foot pain, and unsuitable footwear. It is worth noting that foot examination, including palpation of foot pulses, plays a significant role in identifying risk factors for ulceration in patients with diabetes [29]. It is imperative to direct patients with diabetic foot issues to a multi-disciplinary foot care team. This approach, involving a team of professionals from various disciplines, offers significant benefits to patients and is crucial for achieving optimal management and prevention of complications. The collective efforts of the multi-disciplinary team are focused on restoring and maintaining a lower extremity free from ulcers, with the ultimate objective of preserving functional limbs. Additionally, collaboration among specialists extends to the development of consensus documents and structured educational programs that emphasize the interdisciplinary care of patients with diabetes [36].

The study's findings revealed that, on average, the participants assigned a prioritization score of 54.1 (standard deviation: 11.7) out of a total score of 78 (69.4%) to foot care. This indicates a moderately high level of prioritization for foot care among the study participants. These results emphasize the importance for clinicians to prioritize preventative foot care at an earlier stage in the diabetes care continuum. Additionally, the findings underscore the significance of proactive foot care practices over reactive ones, as such prioritization is crucial in preventing foot ulcers and the subsequent need for amputation [16]. Furthermore, binary logistic regression analysis revealed that healthcare professionals within the age range of 35-44 years, with 5-10 years of experience, employed at private hospitals, managing a higher number of practice clinics per week, and treating a greater number of patients with diabetes in each clinic, exhibited a greater likelihood of prioritizing foot care in their clinical practices. These demographic findings suggest that physicians with more experience and exposure to diabetic patients are more likely to possess awareness regarding foot care and prioritize its implementation in their practice. This practice, encompassing preventive measures, patient and staff education, multidisciplinary treatment of foot complications, and close monitoring, has been shown to be highly effective in reducing the rate of amputations and other complications associated with diabetic foot conditions [37,38].

This research contributes to the comprehension of diabetic foot care in the context of Saudi Arabia, highlighting the pressing need for enhanced prioritizing. It is recommended that policymakers take into account the implementation of focused educational programs for healthcare practitioners, the establishment of standardized foot screening protocols, and the integration of continuous glucose monitoring technologies. Healthcare professionals stand to gain advantages from receiving specialized training, adhering to standards for conducting routine foot inspections, and enhancing their knowledge of the most recent advancements in diabetic foot care. The use of these approaches has the potential to greatly improve patient outcomes, decrease complications, and increase diabetic foot care on a national scale.

This study has limitations. The use of an online survey cross-sectional study prevented us from following up with the study participants and examining causality across the study variables. Besides, it is susceptible to selection bias (which is a phenomenon in which the individuals chosen for a study do not accurately represent the broader population from which they were selected), as not all study participants are users of social media platforms. In addition, all self-administered questionnaires are prone to recall and social desirability bias (which is a phenomenon in which survey participants tend to provide responses that are socially acceptable or desirable rather than providing genuine or correct information). Therefore, researchers should interpret our study findings carefully.

## Conclusions

The prioritization of foot care among healthcare practitioners in Saudi Arabia is assessed to be moderately high. Healthcare professionals within the age range of 35-44 years, possessing 5-10 years of professional experience, employed at private hospitals, managing a higher number of practice clinics per week, and attending to a greater number of patients with diabetes in each clinic demonstrated a higher likelihood of prioritizing foot care within their respective practices. Policymakers should consider the introduction of targeted training initiatives for healthcare professionals, the establishment of standardized protocols for foot screening, and the incorporation of continuous glucose monitoring devices. More detailed, site-technique-based studies can be planned in the future for an even better understanding of diabetic feet. Such a study can be implemented within hospital settings by observing real-world practices related to foot care prioritization. This will provide evidence-based findings, which will help in optimizing healthcare practices and improving patients' health.
